# Unlocking New Bioactive Peptides from Coffee Pulp: A Metagenomics and AI-Driven Discovery Paradigm

**DOI:** 10.3390/foods14213682

**Published:** 2025-10-29

**Authors:** Yamil Liscano, Nicolás Caicedo, Jose Oñate-Garzón

**Affiliations:** 1 Grupo de Investigación en Salud Integral (GISI), Departamento Facultad de Salud, Universidad Santiago de Cali, Cali 760035, Colombia; 2 Grupo de Investigación en Química y Biología (QUIBIO), Departamento Facultad de Ciencias Básicas, Universidad Santiago de Cali, Cali 760035, Colombia; nicolas.caicedo01@usc.edu.co (N.C.); jose.onate00@usc.edu.co (J.O.-G.)

**Keywords:** coffee pulp, bioactive peptides, generative artificial intelligence, metagenomics, waste valorization, Colombia

## Abstract

This perspective reframes Colombian coffee pulp from an environmental liability into a strategic asset by proposing a new discovery paradigm. We argue that the pulp’s challenging chemical environment is not a barrier but its key advantage, having acted as a natural evolutionary filter that has sculpted a unique, highly resilient microbiome. Our vision is a technology pipeline that harnesses this natural pre-selection. By converging deep metagenomic data from the pulp’s microbiome with generative artificial intelligence, we can create and validate novel, high-performance bioactive peptides and enzymes that are already pre-optimized for industrial robustness. This approach transcends traditional waste valorization, establishing a new framework for “biointelligence” in action. It offers a strategic roadmap for Colombia to generate knowledge-intensive value chains from its most iconic agricultural product, turning a national challenge into a global opportunity in the bioeconomy.

## 1. Introduction

This perspective argues for a fundamental paradigm shift in agro-industrial valorization: we must move beyond viewing waste streams as mere substrates and instead recognize them as highly curated evolutionary engines. We posit that Colombian coffee pulp, an abundant and environmentally challenging byproduct, represents an ideal model for this new approach. Its intrinsic chemical inhibitors, such as caffeine and polyphenols, have acted as a powerful natural selection filter, sculpting a specialized microbiome pre-adapted for robustness and efficiency [1,2,3,4]. This article proposes that by coupling deep metagenomic sequencing of this unique niche, which grants access to the vast uncultivable majority [5,6], with generative artificial intelligence (AI) and active learning (AL), we can unlock a new discovery pipeline. This integrated strategy bypasses the limitations of traditional bioprospecting by learning the design rules from nature’s survivors, enabling the de novo design of high-performance bioactive peptides and industrial enzymes that are inherently optimized for challenging bioprocessing environments [7,8].

This vision directly addresses the global challenge of building a sustainable circular bioeconomy [9], which demands transforming environmental liabilities into economic assets. The historical transformation of cheese whey from a costly waste into a multi-billion dollar industry via membrane technology serves as a powerful precedent [10,11,12]; it proved that a single technological catalyst can unlock the immense value latent in an agro-industrial byproduct. We argue that for data-rich bioresources like coffee pulp, the catalytic convergence of genomics and AI is the modern equivalent of that technological shift. For a nation like Colombia, leveraging its most iconic agricultural byproduct in this manner is a strategic imperative, paving the way for a knowledge-intensive bioeconomy built upon its unique biodiversity [13,14].

Our focus on bioactive peptides and industrial enzymes is deliberate. Bioactive peptides, short protein fragments with antimicrobial, antioxidant, or antihypertensive functions, are at the forefront of the functional food and pharmaceutical industries, a market driven by a consumer shift towards preventive health [15,16,17,18,19,20,21]. Simultaneously, novel enzymes tolerant to inhibitors are critical for advancing a greener, more efficient biomanufacturing sector. By targeting these high-margin molecular classes, our proposed paradigm shifts the goal from low-value waste management to high-value, data-driven bioprospecting and molecular design, creating a resilient new value chain for the coffee industry.

## 2. Colombian Coffee Pulp as a Bioresource: A National Challenge and a Unique Niche

The magnitude of coffee pulp production in Colombia is staggering and underscores the urgency of this challenge. As one of the world’s leading coffee producers, the country generates massive volumes of this byproduct, which constitutes a significant fraction of the fruit, representing between 29% and 43% of its total weight [22,23]. In 2023 alone, Colombian coffee production exceeded 11.3 million 60 kg bags [24], translating to more than 1.6 million tons of coffee pulp annually. The inadequate management of this wet, rapidly decomposing biomass leads to severe environmental contamination. Its leachates are characterized by a very high Biochemical Oxygen Demand and Chemical Oxygen Demand, which drastically reduce dissolved oxygen in water bodies and cause widespread aquatic death [25,26]. Furthermore, the leaching of compounds such as phenols and caffeine causes severe acidification of soils, disrupting terrestrial ecosystems and threatening water security for local communities. This environmental burden transforms what should be a valuable bioresource into a significant national-scale liability [23,27].

However, this same massive scale presents a strategic advantage. Unlike other geographically dispersed agricultural residues, coffee pulp production is inherently concentrated at farms and processing facilities. This means the residual raw material is already co-located and generated consistently, dramatically reducing logistical barriers for collection and processing. This characteristic represents a key differentiator compared to other biorefinery models that often face high biomass supply chain costs [28,29,30]. Therefore, while the scale of coffee pulp production creates an environmental crisis, it also transforms the material into an ideal, concentrated, and consistent raw material for a potential bioindustry, as detailed in Table 1.

Furthermore, a critical analysis demands acknowledging the significant economic barriers to its valorization. The pulp’s high moisture content (80–85%) inflates transportation costs and requires energy-intensive dehydration for many conversion pathways [31,32]. The capital expenditure required to build and operate advanced biorefineries represents a formidable obstacle, particularly in rural economies. These economic realities explain why previous valorization efforts have often focused on low-margin products like compost or animal feed, which struggle to justify the initial investment [33,34]. Our perspective confronts this challenge directly by arguing that the only economically viable path is to target high-value, low-volume bioproducts. By focusing on bioactive peptides and specialty enzymes, molecules with market prices orders of magnitude higher than bulk commodities, the high upfront costs of advanced bioprocessing can be justified, creating a truly profitable and sustainable value chain.

Coffee pulp’s chemical composition presents a fascinating duality of opportunity and challenge. It is important to acknowledge, however, that the exact composition can vary significantly based on factors such as coffee variety, geographical region, and specific processing conditions [35]. On one hand, it serves as an exceptionally rich substrate for biotechnological processes. It contains high carbohydrate concentrations (45–89%) and significant protein amounts (4–12%), along with lipids and minerals [1]. This composition makes it an ideal fermentation medium capable of supporting vigorous microbial growth for producing a wide range of value-added products, such as industrial enzymes [36]. Conversely, the pulp is intrinsically recalcitrant due to compounds that are known bioprocess inhibitors, including high caffeine concentrations (up to 1.3%) and elevated loads of tannins and other polyphenols [1,37]. In this context, inhibitors are chemical substances that interfere with or slow down enzymatic reactions and microbial growth, which represents a challenge for conventional fermentation processes [37,38]. These compounds hinder enzymatic hydrolysis and microbial growth in conventional systems, while their high moisture content (80–85%) presents challenges for thermochemical conversion technologies [31].

This duality precisely makes coffee pulp such a promising target for advanced bioprospecting. While inhibitors represent obstacles for traditional biorefinery approaches, they have acted as powerful natural selection forces in the microenvironment of fermenting pulp piles. Microorganisms that have thrived in this niche have evolved robust metabolic mechanisms to tolerate, and often metabolize, these compounds. This “hostile” environment is not a raw material defect; it is the source of its unique scientific value. It contains a treasure trove of biological solutions, inhibitor-tolerant enzymes, unique degradation pathways, and bioactive peptides, pre-selected by nature. Rather than viewing inhibitors as problems to eliminate, this approach considers them the key to finding superior biological solutions [1,28,39].

The pursuit of high-technology valorization for coffee pulp transcends mere academic exercise; it represents tangible implementation of Colombia’s highest-level scientific and economic policy. The 2020 National Bioeconomy Strategy and the 2019 “Misión de Sabios” recommendations constitute the country’s roadmap for knowledge-based sustainable development [14]. These documents explicitly call for moving beyond raw material exports and utilizing science to add value to the nation’s unique biodiversity. The strategy introduces the concept of “biointelligence,” defined as leveraging species’ genomic potential to discover and develop high-value compounds [13]. A research program focused on metagenomic mining of coffee pulp microbiome and AI-mediated biomolecule design embodies biointelligence in action. By focusing efforts on a byproduct from Colombia’s most emblematic industry, this creates a powerful narrative that resonates with national identity while positioning the country to capitalize on global market opportunities, fostering an innovation ecosystem around its biological resources.

## 3. The High-Value Frontier: From Bulk Products to Bioactive Molecules

The proposed valorization paradigm deliberately moves away from low-margin, high-volume products such as compost or animal feed, which, while useful, fail to capture coffee pulp’s true biochemical potential [40]. Instead, the approach centers on extracting and designing two classes of high-value molecules at the forefront of modern biotechnology: bioactive peptides and novel industrial enzymes. Bioactive peptides are short amino acid chains, typically 2 to 20 residues, that remain inactive within their precursor protein sequence. Once released through enzymatic hydrolysis, as occurs during fermentation, they can exert specific and beneficial physiological functions [15]. These activities are diverse and highly sought after, including antimicrobial, antioxidant, antihypertensive, and immunomodulatory effects [2]. Their natural origin and high specificity make them ideal candidates for use in functional foods, dietary supplements, and pharmaceutical products [15]. Meanwhile, industrial enzymes are protein biocatalysts that drive chemical reactions with high efficiency and specificity. Discovering new enzymes, such as cellulases, proteases, or lipases, with enhanced properties like greater thermostability or inhibitor resistance is fundamental for developing more sustainable and profitable industrial processes [36]. An enzyme capable of efficiently degrading biomass in the presence of inhibitors found in coffee pulp would represent an immensely valuable product for the biofuels industry and green chemistry. By establishing these two molecule types as primary targets, the approach shifts from simple waste management to high-level bioprospecting and molecular design.

The economic justification for this pursuit rests on observable market dynamics and evolving consumer preferences. The global bioactive peptides market demonstrates considerable expansion potential, driven by a fundamental shift in consumer behavior toward preventive healthcare and wellness-centered lifestyles. With the global bioactive protein and peptides market projected to reach around USD88.3 billion by the end of 2027, growing at a Compound Annual Growth Rate (CAGR) of 8.2% during the forecast period [41], the economic incentive to valorize a protein-rich waste stream is significant. This trend manifests as increasing demand for functional foods and natural ingredients that offer health benefits beyond basic nutrition [3,4]. Bioactive peptides, with their demonstrated functions, fit perfectly into this megatrend. Crucially for this proposal, plant-based sources represent a dominant and rapidly growing segment within this market, due to both consumer preference and extraction profitability. Coffee pulp, as a protein-rich agro-industrial residue, positions itself as an ideal, low-cost raw material for accessing this lucrative market. This solid business case provides powerful economic “pull” that justifies investment in the proposed high-technology discovery pipeline, demonstrating that the outlined scientific vision has a viable path toward commercialization and creation of a new, valuable value chain for Colombia’s coffee industry [42,43,44].

While these successful precedents are valuable, a strategic analysis reveals critical distinctions that position our proposal as a significant leap forward. Firstly, the feedstocks used, such as okara, banana, and mango pulp, are biochemically benign compared to coffee pulp. Our central thesis is that the pulp’s intrinsically hostile environment, rich in inhibitors like caffeine and polyphenols, is not a drawback but a unique advantage, having pre-selected for a microbiome with novel, highly robust biomolecules not found in these other substrates. Secondly, the discovery methods employed in these examples, such as direct enzymatic hydrolysis [45,46] or standard fermentation [47], are designed to extract what is already known to be present. Our proposed pipeline, leveraging metagenomics and generative AI, transcends this limitation. It is not an extraction tool, but a de novo design engine capable of generating completely novel molecules based on the unique genetic information mined from the pulp’s microbiome. This combination of a uniquely selective feedstock and an advanced discovery engine provides a pathway to first-in-class biomolecules, moving beyond the incremental improvements offered by conventional approaches.

Bioactive peptides are found in complex mixtures at low concentrations, making it essential to use appropriate methods for their extraction and structural characterization. They can be generated from the fermentation or enzymatic hydrolysis of protein precursors or by the transcriptional activation of genes [48]. For the identification of the primary structures of bioactive peptides in mixtures like fruit pulp, samples must be fractionated into simpler sub-samples using high-performance liquid chromatography (HPLC). This technique uses polar/non-polar solvents under a concentration gradient to help move the analyte along the stationary phase, which is typically hydrophobic due to the functionalization of silica with silane of a varied number of carbons [49,50].

Subsequently, the separated fractions are analyzed by mass spectrometry, either by incorporating them into the Matrix-assisted laser desorption/ionization (MALDI) technique or through extraction by solvent evaporation, such as electrospray ionization (ESI) [51,52]. These techniques are used to generate both positive and negative ions; however, positive ionization [M + H]^+^ is considered more suitable for peptide analysis, as the positive charge of the peptide can be stabilized by the basic amino acid residues, which are protonated under ionization conditions [2]. After collecting the mass spectra, peptides are identified by searching databases, comparing the tandem/experimental spectra with the theoretical spectra from a library of known peptides. The protein sequence databases commonly used are X!Tandem, SEQUEST, and Mascot [2,53]. A complementary approach, often referred to as proteomics, involves the large-scale study of proteins and peptides to understand their functions and structures. With the advancement of AI, tools like AlphaFold and ESMFold have revolutionized this field by accurately predicting the three-dimensional structure of a peptide from its amino acid sequence [54,55]. This structural information is invaluable for predicting a molecule’s function, binding sites, and potential bioactivity in silico, significantly accelerating the design and validation process before experimental work begins. This integration of advanced proteomic analysis with AI-driven structural prediction is a cornerstone of the proposed pipeline.

## 4. A New Discovery Paradigm: Integrating Metagenomics and Generative AI

The proposed discovery engine addresses a fundamental limitation of classical microbiology: the inability to cultivate the vast majority of microorganisms in the laboratory. Researchers estimate that over 99% of microbes present in an environmental setting are “uncultivable” with standard techniques, meaning their vast genetic and metabolic potential remains inaccessible to traditional screening methods [56]. This microbial “dark universe” represents an unexplored frontier for biomolecule discovery. Metagenomics is the technology that allows us to penetrate this darkness; by directly sequencing the collective DNA of an environmental sample, it completely bypasses the need for cultivation [6].

The power of applying metagenomics to the coffee ecosystem has already been demonstrated across Latin America, where it has been used to map the complex microbial communities driving fermentation. Table 2 summarizes several of these key studies, revealing the vast, previously hidden diversity and establishing a clear link between regional microbial signatures, processing methods, and environmental factors.

Analysis of these studies reveals consistent patterns. A clear microbial succession is common in tropical climates, where diverse initial communities give way to dominant Lactic Acid Bacteria (LAB) and yeasts. The profound influence of terroir is also evident, with factors like altitude and climate significantly shaping the microbial consortia. Methodologically, the shift towards shotgun metagenomics has deepened the analysis from simple taxonomic lists to functional genomics, allowing the reconstruction of genomes and metabolic pathways. However, this synthesis exposes a critical research gap. With the vast majority of studies focusing on the fermenting bean or its wastewater, the coffee pulp, the most abundant and chemically unique byproduct, remains a largely unexplored frontier for systematic bioprospecting [5]. It is important to note that while metagenomics reveals vast genetic potential, complementary methods such as culturomics can be crucial for isolating and characterizing viable microorganisms and their specific phenotypes. Culturomics, by using high-throughput culture conditions, can be essential for targeted strain isolation for future bioprocess development or for finding new producer organisms. This makes applying a multi-faceted bioprospecting approach to the pulp particularly promising [63,64]. As established, the coffee pulp pile represents an “extreme” niche characterized by unique selective pressures (low pH, high inhibitor concentrations), which has driven the evolution of a highly specialized microbiome. Therefore, metagenomic bioprospecting of this environment is not a random search, but a targeted screening for genes already optimized by nature to function effectively in coffee pulp’s specific matrix [65].

If metagenomics is the explorer discovering nature’s existing treasures, generative AI is the artisan capable of creating completely new and optimized masterpieces. The discovery engine’s second stage harnesses the power of deep generative models, such as VAEs and GANs, to transcend natural discovery limits and enter the realm of de novo design [7]. These AI models learn the underlying patterns and “grammar” of large databases of known peptide sequences and, once trained, can generate virtual libraries of millions of completely new sequences with high probability of possessing desired biological activity [66]. Advanced architectures like MPOGAN even incorporate feedback loops to simultaneously optimize multiple properties, such as high antimicrobial activity and low cytotoxicity [8]. When using generative AI for decision-making, it is crucial to consider its limitations. For example, these algorithms have a tendency to create non-existent references, which is a clear problem that can lead to misleading or inaccurate data that is difficult to distinguish from real information [67], Furthermore, validating the information can be challenging [68]. Therefore, a cautious and verifiable method, such as a bibliographical search, would be a good way to avoid biases in the reported information.

**Figure 1:**
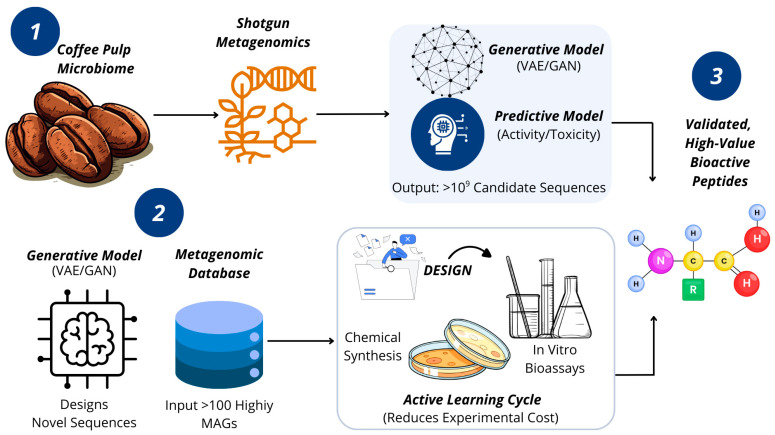
The Integrated Discovery Pipeline. This conceptual model illustrates how our pipeline overcomes key bottlenecks. Metagenomic mapping bypasses biochemical inhibitors, feeding data to a central AI Transformation Engine. This engine designs novel biomolecules, and an iterative Active Learning Cycle intelligently guides experimental validation, accelerating the discovery of high-value peptides and enzymes. Source: own elaboration, created with Canva (https://www.canva.com/ accessed on 21 September 2025).

The true innovation of the proposed pipeline, schematized in Figure 1, resides in the synergy between these two technologies. Metagenomics alone can only find what already exists, while generative AI trained solely on generic databases may not produce molecules optimized for specific industrial contexts. Integrating both overcomes these limitations: metagenomics discovers a unique, contextually relevant dataset of robust biomolecules from coffee pulp microbiome, and this dataset is used to train or fine-tune a generative AI model.

The resulting AI model learns specifically the “design rules” of biomolecules that thrive in coffee pulp environments, generating novel sequences that are not only functional but pre-optimized for robustness and inhibitor tolerance. This self-reinforcing discovery loop, where natural exploration feeds digital creation, forms the core of the new paradigm, whose advantages over traditional approaches are summarized in Table 3.

## 5. Navigating the Labyrinth: Overcoming Key Bottlenecks

A credible perspective must address the most significant challenges head-on. On the path from concept to realization, the proposed valorization pipeline faces two main obstacles: the biochemical barrier inherent to the raw material itself and the economic chasm between computational generation and experimental validation. This section acknowledges these bottlenecks and presents integrated technological solutions designed to overcome them, summarized in Table 4.

The first challenge lies in coffee pulp’s chemical composition. As detailed, the pulp contains a potent mixture of bioprocess inhibitors, primarily caffeine and polyphenols like tannins [1]. These compounds are known to severely hinder enzymatic hydrolysis and microbial growth in conventional fermentation systems. In a traditional biorefinery approach, the presence of these inhibitors would require costly pretreatment and detoxification steps to remove offensive compounds before the biomass could be efficiently processed [69]. The solution to this biochemical barrier, however, does not reside in additional chemical pretreatment, but in the bioprospecting strategy itself. The “Hostile Environment Advantage,” as argued earlier, converts this challenge into an opportunity. By directing our search for enzymes and peptides to the microbiome that has already evolved for generations within coffee pulp piles, we are specifically selecting microorganisms that have developed natural resistance. The biological tools we discover are already adapted for the task [5]. Rather than fighting against pulp chemistry, we leverage it as a natural selection filter to discover superior biocatalysts.

The second and perhaps most formidable bottleneck represents an endemic problem of the modern biomolecule discovery era. Advances in AI and high-performance computing have created radical asymmetry: we can now design molecular candidates in silico at rates that exceed by orders of magnitude our capacity to synthesize and test them in the laboratory [7]. Generative AI models can produce millions of promising sequences in hours, but each experimental test requires time, expensive reagents, and specialized labor. This mismatch creates the “experimental validation bottleneck,” a chasm between computational design and real-world testing that represents the greatest barrier to commercialization [7]. Brute-force screening is simply unfeasible.

The solution to this chasm is AL, a machine learning paradigm designed precisely for resource-limited scenarios. AL represents a form of intelligent experimentation. Rather than testing random candidates or only the “best” predicted ones, AL iteratively selects data points expected to provide maximum information for improving the predictive model. The process functions as a closed feedback loop, often termed the Design-Build-Test-Learn (DBTL) cycle, where experimental results from a small batch of candidates are used to retrain and improve the model, which then selects the next most informative batch. This iterative approach allows the model to learn the complex sequence-activity relationship much more efficiently, drastically reducing necessary experimental effort [70]. AL is therefore the missing link that makes generative AI-driven discovery pipelines economically viable. It transforms the process from intractable screening to strategic, intelligent search, closing the gap between computational imagination and physical reality.

While the proposed pipeline addresses key technical bottlenecks, a credible perspective must confront the formidable real-world hurdles to commercialization. The analysis of these limitations must move beyond generalities and detail the specific regulatory routes, economic barriers, and risks of bias in AI.

From a regulatory perspective, achieving market approval for peptides from a novel source like coffee pulp is a multi-year, multi-million-dollar challenge. In the United States, this would likely require compiling a comprehensive dossier to achieve Generally Recognized as Safe (GRAS) status from the Food and Drug Administration (FDA), a process demanding extensive toxicological and efficacy data. In Europe, it would fall under the European Food Safety Authority’s (EFSA) stringent “Novel Food” regulation, which requires a rigorous safety assessment before market authorization [71,72,73]. Gaining approval for a complex, microbially derived product from a waste stream would undoubtedly face heightened scrutiny.

From an economic perspective, the “valley of death” between laboratory-scale success and profitable industrial production is the primary obstacle. Beyond the initial high capital expenditure for bioreactors, the majority of biomanufacturing costs often lie in downstream processing, the complex and expensive steps required to purify the target peptide to commercial grade [1,74]. Furthermore, ensuring a consistent and stable supply chain for a variable agricultural residue like coffee pulp poses significant logistical challenges that could impact batch-to-batch product quality and yield.

A critical view of the risks inherent to generative AI is essential. While powerful, these models are not infallible. They can inherit biases from the public databases on which they are trained, potentially limiting their ability to generate truly novel sequences optimized for the unique coffee pulp environment [75]. There is also a tangible risk of generating non-functional peptides or, in a worst-case scenario, sequences with unintended toxicity. This underscores that our proposed AL loop is not merely an accelerator but a crucial safety and validation mechanism, as in silico promise must always be subject to rigorous experimental verification [76,77].

## 6. Roadmap and Future Vision for Biointelligence in Colombia

Our proposed model transcends traditional academic boundaries and requires a synergistic consortium for its success. This is a multi-institutional, multi-sector collaboration that unites the scientific and technological expertise of the proposed pipeline with the economic and strategic acumen of industry. The proposed consortium will include key actors from the public, private, and academic sectors. This would involve governmental agencies (e.g., Minciencias) to provide a regulatory framework and strategic funding; national research centers (e.g., Cenicafé, Agrosavia) to contribute foundational knowledge and technical expertise in coffee processing; private sector companies (e.g., coffee producers, food and nutraceutical companies) to provide market insights and a direct path to commercialization; and academic partners to drive fundamental research and train the next generation of bioprospecting scientists. This collaborative structure is essential to manage the complex knowledge flows, regulatory challenges, and investment requirements inherent in a disruptive project of this scale.

For this vision to materialize, a structured research program is required that moves beyond a conceptual journey and establishes a credible implementation pathway. We have therefore structured our roadmap according to the widely adopted Technology Readiness Level (TRL) scale, defining three core phases that advance the concept from basic research to a validated pilot-scale process [78,79]. Each phase integrates the key technological components into a cohesive pipeline and is defined by clear objectives, activities, and measurable milestones.

The first phase, Proof of Concept & Viability (TRL 1–3), aims to validate the central hypothesis that the coffee pulp microbiome contains unique, exploitable genetic potential. Key activities combine Bioresource Mapping and In Silico Bioprospecting, involving the systematic collection and deep shotgun sequencing of coffee pulp samples to construct high-quality Metagenome-Assembled Genomes (MAGs) [6]. Advanced bioinformatics pipelines will then be used to annotate these MAGs and identify a catalog of “genes of interest,” such as inhibitor-tolerant enzymes and novel bioactive peptide pathways [56]. The key milestone for this phase is the creation of a curated database of the Colombian coffee pulp microbiome, containing a critical mass of high-quality MAGs and a validated list of genetic targets for the next phase.

The second phase, Technology Development & Lab-Scale Validation (TRL 4–5), is designed to demonstrate that the integrated pipeline can successfully generate and validate novel peptide candidates with demonstrable bioactivity. Its activities integrate Generative Model Training and Active Learning (AL)-Guided Validation. The genetic data from Phase I will be used to train a generative AI model to design a virtual library of novel peptides [7]. Subsequently, the AL loop will be implemented to intelligently select, synthesize, and test small batches of candidates, with the experimental results used to iteratively retrain and improve the AI model, rapidly converging on highly active leads [70]. The key milestone for this phase will be the experimental validation of at least two lead peptide candidates with significant bioactivity and low cytotoxicity in laboratory assays.

The third phase, Demonstration & Pilot-Scale Optimization (TRL 6–7), will focus on producing the validated lead peptides in an industrially relevant microbial host to demonstrate a viable bioprocess at a pilot scale. The primary activities for this Optimization and Scale-up phase will be the heterologous expression of the lead peptides in a robust microbial chassis, such as *Saccharomyces cerevisiae*. Fermentation processes will be optimized in laboratory-scale bioreactors, using coffee pulp hydrolysate as a potential feedstock component to demonstrate circularity [30]. The key milestone will be the successful demonstration of peptide production in a pilot-scale bioreactor, achieving predefined targets for titer, yield, and productivity.

**Figure 2:**
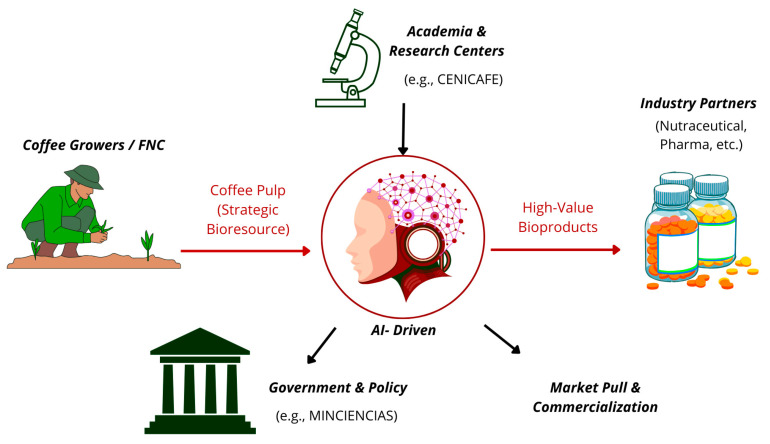
Circular Bioeconomy Model for Coffee Pulp. This schematic illustrates a cycle where pulp, supplied by coffee growers, is analyzed to extract high-value biological data. An AI-driven platform processes this information to design bioproducts, which are then fabricated using advanced biomanufacturing. The model is supported by academia (research and talent) and government (policy and support), generating additional income for producers and converting an agricultural residue into a strategic resource. This entire model exemplifies a sustainable, circular bioeconomy framework. Source: own elaboration, created with Canva (https://www.canva.com/ accessed on 21 September 2025).

This integrated pipeline transforms coffee pulp from a simple agro-industrial residue into the foundation of an advanced biomanufacturing ecosystem, where high-value biological data extracted from the unique coffee pulp microbiome feeds an AI-powered transformation core that generates novel bioactive peptides and enzymes for diverse applications in pharmaceutical, nutraceutical, and green chemistry sectors, as conceptualized in Figure 2. This workflow represents a paradigmatic shift from traditional waste management to strategic bioresource valorization, positioning coffee pulp as a cornerstone of Colombia’s emerging bioeconomy.

**Figure 3:**
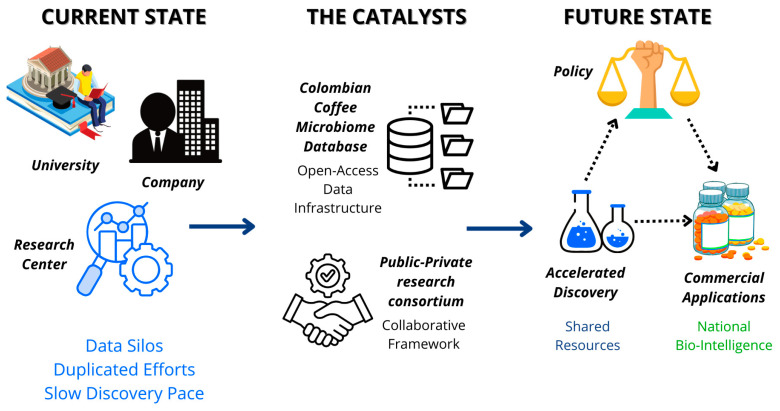
Strategic Framework for Colombian Coffee Bioeconomy Implementation. This diagram illustrates the transformation from fragmented research efforts to an integrated innovation ecosystem. The framework centers on two strategic initiatives: establishing a Colombian Coffee Microbiome Database (providing open-access data infrastructure) and forming a Research Consortium (integrating academia, research centers, and industry). This collaborative approach catalyzes the transition from duplicated, slow discovery processes to an accelerated discovery pace with enhanced collaboration and commercial applications, ultimately positioning Colombia as a leader in biointelligent valorization of agricultural resources. Source: own elaboration, created with Canva (https://www.canva.com/ accessed on 21 September 2025).

Long-term success of this vision demands building a collaborative innovation ecosystem. To catalyze it, two strategic initiatives are proposed. First, creating an open-access Colombian Coffee Microbiome Database. Shared, pre-competitive data infrastructure is fundamental in the AI-driven biology era, as it would foster collaboration, avoid effort duplication, and accelerate discovery pace at a national scale [7]. Second, forming a public–private research consortium that brings together academia, national research centers like Cenicafé, with their deep knowledge of coffee and its byproducts [24], and industry. This tripartite collaboration model is essential to ensure fundamental research translates into commercial applications, aligning with the model proposed in Colombia’s National Bioeconomy Strategy [14]. These strategic initiatives work synergistically to transform the current landscape from fragmented research efforts with duplicated work and slow discovery progress to an accelerated discovery ecosystem where collaborative frameworks and commercial applications can flourish, as illustrated in Figure 3.

The perspective presented here advocates for a fundamental redefinition of the coffee industry in the 21st century. The future of coffee cultivation in a prosperous bioeconomy cannot depend solely on the bean; it must embrace integral valorization of the fruit and the microbial universe that accompanies it. We envision a future where Colombia’s coffee farms not only produce a world-renowned product but also serve as sources of unique biological data that will become raw material for a sophisticated AI-driven biomanufacturing industry. This approach transforms coffee pulp from an environmental burden into a strategic national asset, aligns Colombia’s most iconic industry with the frontiers of science and technology, and creates new, resilient value chains. In doing so, it not only solves a waste problem but also positions Colombia as a global leader in sustainable and intelligent application of its incomparable biodiversity, thus fulfilling the bold and necessary promise of its “Misión de Sabios” [13].

## 7. Conclusions

The paradigm presented in this perspective offers a fundamental redefinition of coffee pulp, recasting it from an environmental liability into a strategic bioresource rich with data. We argue that the convergence of metagenomic mining and generative AI on this unique, naturally selective niche represents a paradigm shift in bioprospecting. By treating the pulp’s challenging chemical environment as a feature, not a flaw, we can unlock an evolutionary shortcut to discovering and designing novel, high-performance biomolecules that are pre-optimized for industrial robustness.

However, the path to realizing this vision is not without significant challenges. As we have discussed, formidable regulatory hurdles in key markets, substantial economic barriers to scaling biomanufacturing, and the inherent technological risks of AI-driven discovery must be proactively managed. Overcoming these constraints is not a trivial task and will require a concerted, multi-stakeholder effort that aligns scientific innovation with strategic investment and supportive public policy.

The immediate priority, therefore, is to validate the foundational hypothesis of this work by initiating the first phase of our proposed roadmap (TRL 1–3): the systematic bioresource mapping and in silico bioprospecting of the Colombian coffee pulp microbiome. Success in this initial phase will provide the critical, context-rich data needed to fuel the AI-driven discovery engine. Ultimately, this approach of treating agricultural byproducts as curated evolutionary datasets has the potential to become a blueprint for the broader circular bioeconomy. If successful, it could position Colombia not just as a producer of premium coffee, but as a global leader in the data-driven valorization of its immense biodiversity.

## Figures and Tables

**Table 1:** Quantitative and Qualitative Profile of Colombian Coffee Pulp as Raw Material for Biorefinery.

Parameter	Value/Range	Implication for Bioprocessing	Reference(s)
Annual Generation (Colombia)	>1,600,000 tons (2023)	Concentrated, large-scale raw material source	Federación Nacional de Cafeteros de Colombia (2023) [24]
% of Cherry Weight	29–43% (wet basis)	Main byproduct, available in predictable quantities	Fernández Cortés et al. (2020) [23]
Moisture Content	80–85%	Requires wet processing or energy-intensive drying	Setiawan et al. (2025) [31]
Protein	4–12% (dry basis)	Potential nitrogen source for fermentation; peptide raw material	Hua et al. (2023) [1]
Total Carbohydrates	45–89% (dry basis)	Primary carbon source for microbial growth	Hua et al. (2023) [1]
Lignin	~20–26%	Recalcitrant component; potential source of aromatic compounds	Arango-Agudelo et al. (2023) [22]
Caffeine	~1.3% (dry basis)	Known inhibitor of many microorganisms; selective pressure	Hua et al. (2023) [1]
Total Phenols/Tannins	High content	Hydrolytic enzyme inhibitors; selective pressure; antioxidant source	Hua et al. (2023) [1]

**Table 2:** Summary of coffee fermentation microbiome studies across Latin America.

Reference and Country	Processing and Sequencing	Key Microbes Identified	Main Findings	Biotechnological Potential
Martínez et al. (2021), Brazil (Caparaó) [57]	Natural, Self-Induced Anaerobic Fermentation (SIAF); Illumina MiSeq (16S V3–V4, ITS)	Bacteria: Gluconobacter, Weissella (800–1000 m); Sphingomonas, Methylobacterium (1200–1400 m). Fungi: Cystofilobasidium dominant at all altitudes.	Altitude strongly shapes microbiota and volatile profile. Low altitudes → higher bacterial richness and alcohols. High altitudes → more esters, aldehydes, organic acids. First report of Sphingomonas and Nakamurella in natural coffee.	Yeasts (*M. caribbica*, *W. anomalus*) proposed as starters. Supports terroir-driven quality control.
de Carvalho Neto et al. (2018), Brazil (Cerrado Mineiro) [58]	Wet fermentation; Illumina 16S (V4)	LAB dominated (>97%), esp. Leuconostoc, Lactococcus. New: Fructobacillus, Pseudonocardia, Pedobacter, Sphingomonas.	>80 bacterial genera identified (diversity underestimated before). Clear microbial succession with LAB dominance. High lactic acid linked to LAB abundance.	*Fructobacillus* may reduce residual sugars. Newly reported genera may serve as functional starters.
Pothakos et al. (2020), Ecuador (Nanegal) [59]	Wet fermentation; Shotgun Metagenomics	Succession: 1. Early: Tatumella, Acetobacter, Hanseniaspora. 2. Mid: *Leuc. pseudomesenteroides*. 3. Late: Acid-tolerant LAB (*L. vaccinostercus*, *L. brevis*).	>150 species detected. 22 near-complete bacterial genomes reconstructed. Functional shift: plant cell-wall degradation → sugar metabolism by LAB.	Novel hexose-phosphate transport in *Leuc. pseudomesenteroides*. Genomic inventory enables targeted starter design (e.g., GABA producers).
de Oliveira Junqueira et al. (2019), Colombia (Nariño) [60]	Traditional wet method; Illumina (16S and 18S)	Bacteria: >160 genera, LAB > 60%. Fungi: *Pichia nakasei* dominant.	First report of Colombian coffee microbiome. 56 new genera reported. Strong terroir influence (soil, insects, humans). Lactic acid linked to LAB; acetaldehyde to *Pichia*.	Rich diversity may serve as flavor modulation. Endemic microbes as origin markers for certification.
Cruz-O’Byrne et al. (2021), Colombia (Sierra Nevada) [61]	Wet fermentation; Illumina MiSeq (16S V3–V4, ITS2)	Bacteria: LAB (*Leuconostoc*), AAB (*Acetobacter*). Fungi: *Kazachstania* (first dominant report in coffee mucilage), others.	Highest microbial richness reported (695 bacterial, 156 fungal genera). Specialty cup quality correlated with fungal richness, *Pichia*, *Pseudomonas*. Network analysis: LAB–AAB and yeast–AAB co-occurrence.	Balance of LAB–AAB–yeasts key for quality. High native diversity useful for regional starter cultures.
Vale et al. (2024), Brazil (Santa Catarina) [62]	Wet fermentation in humid subtropical climate; Illumina (16S, ITS)	Bacteria: Enterobacteriaceae (Enterobacter, Pantoea, Kluyvera). Fungi: Filamentous (Fusarium, Cladosporium, Penicillium). Notably absent: LAB, yeasts.	Microbiota differs from tropical regions. Final coffee scored as specialty (80.8) but with low complexity. High residual sugars → inefficient fermentation.	Highlights need for tailored starters (LAB, yeasts) in non-traditional regions. Relevant for adapting coffee to climate change.

Abbreviations: LAB: Lactic Acid Bacteria; AAB: Acetic Acid Bacteria; ITS: Internal Transcribed Spacer; SIAF: Self-Induced Anaerobic Fermentation; GABA: Gamma-Aminobutyric Acid.

**Table 3:** Comparative Analysis of Bioactive Peptide Discovery Paradigms.

Paradigm	Primary Mechanism	Key Strength	Primary Limitation	Reference(s)
Traditional Discovery (Screening-Based)	Laboratory screening of natural extracts	Finds naturally occurring molecules with proven activity	Slow, low throughput, expensive; ignores non-cultivable diversity	Purohit et al. (2024) [2]
Functional Metagenomics	Screening of environmental DNA gene libraries without cultivation	Accesses non-cultivable biodiversity; discovers completely new genes/pathways	Only finds what already exists; functional screening can be laborious	Popovic et al. (2015) [5]
Generative AI (Independent)	De novo sequence generation from learning existing databases	Vast design space, high novelty, capability to optimize properties in silico	“Validation bottleneck”: cost of testing generated candidates is prohibitive	Dean & Walper (2020) [66]
Proposed Integrated Pipeline (Metagenomics + Generative AI + Active Learning)	Iterative AI-guided experimental design, fed by unique metagenomic data	Maximizes discovery efficiency, minimizes experimental cost, combines novelty with biological relevance	Requires complex integration of multiple technologies and interdisciplinary expertise	Ariaeenejad et al. (2024) [56]

**Table 4:** Strategic Mitigation of Key Bottlenecks in an AI-Driven Coffee Pulp Valorization Pipeline.

Identified Bottleneck	Negative Consequence If Unaddressed	Proposed Mitigation Strategy Within Framework	Reference(s)
Raw Material Inhibitors (Caffeine, Tannins)	Low fermentation/hydrolysis yields; need for costly pretreatment	Metagenomic bioprospecting of pulp microbiome to discover inherently inhibitor-tolerant enzymes and metabolic pathways	Popovic et al. (2015) [5]
Experimental Validation Cost	Prohibitive R&D costs; inability to explore AI’s vast design space	Implementation of Active Learning loop to guide synthesis and screening, reducing necessary experiments	Goles et al. (2024) [7]
Data Scarcity for AI Models	Poor-performing AI models or limited generalization capability to truly novel sequences	Creation of coffee microbiome database to provide rich, contextually relevant training data	Liu et al. (2024) [8]
Bioprocess Scalability	Laboratory-scale success that does not translate to viable industrial production	Use of robust, industrially relevant microbial chassis (e.g., *S. cerevisiae*) from the start for heterologous expression	Thaha et al. (2025) [30]

## Data Availability

No new data were created or analyzed in this study. Data sharing is not applicable to this article.

## Abbreviations

The following abbreviations are used in this manuscript:

AI	Artificial Intelligence
AL	Active Learning
CAGR	Compound Annual Growth Rate
DBTL	Design-Build-Test-Learn
ESI	Electrospray Ionization
EFSA	European Food Safety Authority
FDA	Food and Drug Administration
GANs	Generative Adversarial Networks
GRAS	Generally Recognized as Safe
HPLC	High-Performance Liquid Chromatography
MAGs	Metagenome-Assembled Genomes
MALDI	Matrix-Assisted Laser Desorption/Ionization
R&D	Research and Development
TRL	Technology Readiness Level
VAEs	Variational Autoencoders
WPC	Whey Protein Concentrate
WPI	Whey Protein Isolate
